# The Portland Meeting

**Published:** 1905-08

**Authors:** 


### THE PORTLAND MEETING.

We owe the following to the New Orleans *Medical and Surgical Journal:*

The fifty-sixth annual meeting of the American Medical Association has come and gone. It was held at Portland, Oregon, on July 11 to 14, and attained quite a measure of success. The attendance reached nearly 1,800, which was excellent for a place so remote from the centers of population.

The local profession and the people did all in their power to make things enjoyable for their guests, and everybody, notwithstanding a few hardships endured, had a good time. This phase of the proposition can best be described in a nutshell, especially in reference to the feelings of the Portland doctors, but also to those of the strangers, by repeating the story of the girl who had just taken her first toboggan ride in Canada. She said it was the finest thing she had ever experienced in all her life, but she wouldn't try it over again for anything in the world.

The truth of the matter is, that the Portland contingent worked like trojans and the others enjoyed themselves, but the Rose City is too new and not quite large enough to handle, without great effort, a meeting like the A. M. A.

Portland is well-named the Rose City, for the queen of flowers, as tree or bush or vine, abounds everywhere, is delightfully perfumed, and attains enormous size. The city is well laid out, is clean, has good street-car service and possesses an enchanting climate. The registering thermometer on the hotel piazza recorded nothing less than 50° nor more than 70° during the week of the meeting, and only once these extremes, hovering most of the time in the neighborhood of 60°. In the coldest winters the temperature rarely gets as low as 10° above zero.

The House of Delegates was numerously represented, and its meetings well attended. Of the large number of amendments proposed to the by-laws but few were adopted. The only two of importance was one providing for oral nomination of the officers except the treasurer, who is still to be nominated by the trustees, thus doing away with the nominating committee; the other puts the control of all arrangements for the annual session under the control of the Board of Trustees, who may appoint a local committee which shall remain under the control of the Board.

The report of the Trustees showed that the Association is in a flourishing financial condition, with assets of $213,470.

The expected discussion about what was to be considered ethical for doctors to use and journals to advertise, and which promised to be quite warm, was successfully (?) disposed of by the report of the special committee which seriously enunciated the *dictum* that NOSTRUMS and their use should be frowned down and should not be advertised in proper medical journals. That is surely elastic enough for most purposes, and one might well add, "you pays your money and you takes your choice."

The only exciting event was the discussion upon the proposition to publish a directory of *all the licensed practitioners* of the United States and the purchase as a means towards this end of the Standard or Englehardt's Directory. Some did not favor the publication of a general directory, but wanted it limited to the members of the Association; others thought it would be boycotting the poor fellows who were not yet members, but would be induced to become such if they were not left out and only tagged as non-members. Some did not favor the purchase of an inaccurate directory; others thought it a *sine qua non*, as otherwise the Association would have to spend all its money to make up its own directory. It ended by the Trustees having their own sweet will and being authorized to publish a directory of all legal practitioners, and to buy Engelhardt's Directory for a sum which they (the Trustees) did not feel authorized to divulge.

The House elected the following officers; President, William Mayo, of Rochester; First Vice-President, Walter Wyman, of P. H. and M. H. Service; Second Vice-President, K. A. J. Mackenzie, of Portland; Third Vice-President, Eug. Talbot, of Chicago; Fourth Vice-President, E. D. Martin, of New Orleans (we came away with something, anyway); Secretary, Geo. H. Simmons, of Chicago; Treasurer, Frank Billings, of Chicago. The three vacancies on the Board of Trustees, after numerous nominations and an exciting race, were filled by the re-election of the three outgoing incumbents: Montgomery, of Pennsylvania; Johnson, of District of Columbia, and Wright, of Iowa.

As orators on medicine, surgery and state medicine, respectively, were elected F. C. Shattuck, of Boston, Jos. D. Bryant, of New York, and W. H. Sanders, of Alabama.

Boston was selected as the place of meeting in 1906.

The section work was creditable; many good papers were read and the discussions were interesting, but nothing startling was developed. The allied associations held their usual annual sessions and had successful meetings. The principal ones were: the American Uroological Association; the American Medical Editors Association; the Society for the Study and Cure of Inebriety, and the Association of State Medical Journals.

The entertainments were numerous and largely attended, some times too largely for comfort, but that was a natural consequence and everybody made the best of it and all enjoyed themselves more or less. Both Tuesday and Wednesday morning there was a ride to Portland Heights on the trolley, and a magnificent view of the city and vicinity was obtained. Tuesday evening a reception was given at the American Inn (The Inside Inn of the Exposition); this was followed by a special performance of Kiralfy's Carnival of Venice, giving unlimited opportunities for pantomime, ballets and music. Wednesday evening receptions were tendered by Mrs. R. B. Wilson, Mrs. K. A. J. Mackenzie and Mrs. Wm. Jones at their residences, and by Dr. and Mrs. H. W. Coe at the Oregon building in the fair grounds. At all places, pretty women beautifully gowned and surrounded by choice samples from the Rose City's gardens made lovely pictures which will long be remembered. Thursday evening a reception and musicale was given at the "Oaks," a pleasure resort reached by trolley and corresponding to the "West End" of New Orleans.

Friday morning was the last and most enjoyable of the entertainments. As many of the guests as the four boats chartered for the occasion could hold left at 9 o'clock for an excursion up the Columbia river. Those who could not be accommodated went by special train at noon to join the others at Bonneville, where an *al fresco* luncheon was served. They were given the first chance of getting back by boat, so that all had the opportunity of making the trip at least one way by water. It was a delightful outing, and we were all able to witness the scenery, which is unsurpassed in its way, many claiming it to be superior in beauty to that along the Hudson.

Of course, the "Trail," which is synonymous with the "Pike" at St. Louis, and the "Midway" at Chicago, was pretty well patronized, and no doubt many members will have amusing incidents to relate when they reach home again about "Gay Paree" and the "Streets of Cairo."

The addresses of welcome delivered at the opening general meeting were unusually good, and brought out the fact that both the Governor of Oregon and the Mayor of Portland were Democrats, elected in a Republican stronghold. Also, we were glad to find that the mayor was a practicing physician, and good at both lines. He usually is a strict enforcer of the laws, in fact was elected on that platform, but on this occasion had instructed the chief of police to see that no member of the A. M. A. was to be interfered with unless it could not possibly be avoided.

We hope Mayor Lane's orders were not found too hard to follow by Portland's finest, but many of the city's guests, after a shell-fish dinner at the "Quelle," where good Munich Beer is also dispensed, seemed to own the town on the Willamette. By the way, the name of this river is no longer pronounced as when the *Journal* representative went to school. The right way now, and this was told as the way to remember it, is to pronounce it so it will rhyme with "damn it."

By any other pronunciation it would be just as fine, the people that live on its borders just as hospitable, the climate just as heavenly, so that one, at least, does not say like the Canadian girl, for he would be glad to take that toboggan ride again.

---

DR. LEWIS M. GAINES, of Atlanta, has been appointed professor of anatomy and physiology in the medical department of Wake Forest College, at Wake Forest, N. C. Dr. Gaines is the son of Rev. S. H. Gaines, president of Agnes Scott Institute, and is a graduate of Johns Hopkins University.

---

The work of the German Congress of Scientists and Medical Practitioners, which is to be held at Meran from September 24 to 30, is divided into main branches, one of which is devoted wholly to medical science. This branch is subdivided into fifteen sections, as follows: (1) Anatomy, histology, embryology, and physiology; (2) general pathology and morbid anatomy; (3) internal medicine, pharmacology, balneology, and hydrotherapy; (4) history of medicine and of the natural sciences; (5) surgery; (6) obstetrics and gynecology; (7) children's diseases; (8) neurology and psychiatry; (9) ophthalmology; (10) dermatology and syphilology; (11) dental surgery; (12) military hygiene; (13) forensic medicine; (14) hygiene, bacteriology, and tropical hygiene; (15) veterinary surgery.

---

There are over two hundred mineral springs in the State of California.

